# Assessing improvements in emergency department referrals to a hospital-based violence intervention program

**DOI:** 10.1186/s40621-021-00333-x

**Published:** 2021-09-13

**Authors:** Jayda Watkins, Na’il Scoggins, Brooke M. Cheaton, Mark Nimmer, Michael N. Levas, Shannon H. Baumer-Mouradian, Marlene D. Melzer-Lange

**Affiliations:** 1 Pediatrics, Medical College of Wisconsin, Milwaukee, WI 53226 USA; 2 Children’s Wisconsin, Milwaukee, WI 53226 USA

**Keywords:** Youth violence, Violence intervention program, Quality improvement

## Abstract

**Background:**

Youth violence is a major public health concern in the United States. Hospital-based Violence Intervention Programs (HVIPs) are integral in connecting youth sustaining interpersonal violence-related injuries to medical, mental health, and social services. At our pediatric emergency department, our baseline referral rate to the established HVIP was 32.5%. From November 2018–2019, we aimed to increase the percent of eligible patients referred to our HVIP from 32.5 to 70% for patients aged 7–18 years who present to our Level 1 emergency department/trauma center with a violent injury.

**Methods:**

For this quality improvement project, we recorded key aspects of the referral process, such as patient eligibility, who placed referrals, and when referrals were placed in relation to the ED admission. Key stakeholders were interviewed to identify specific interventions. Our key interventions were: 1. Educating providers on eligibility requirements. 2. Encouraging nurses to enter consults at the time of admission. 3. Publishing information about program referrals in the weekly nursing newsletter. 4. Updating social workers on eligibility requirements for the HVIP. We used PDSA cycles to inform our project. Our primary outcome measure was the number of eligible patients referred to our HVIP and measures were analyzed using statistical process control charts.

**Results:**

The HVIP-eligible population had the following demographics: 31.1% female and a mean age 14.3 ± 2.7, 82.6% assaults and 17.4% gunshot wounds. From 11/2018 to 11/2019, there were 78 referrals to the HVIP, out of 167 eligible patients. The referral rate improved from 32.5% pre-interventions to 61.1% post-interventions, showing an 88% increase.

**Conclusion(s):**

We noted an increase in referrals to our HVIP following our interventions that centered on educating, advertising, and encouraging. Future studies will focus on analyzing other aspects of the enrollment process, such as obtaining patient consent.

## Background

Youth violence is a major public health concern in the United States. Hospital-based violence intervention programs (HVIPs) are programs that identify youth at risk of repeat interpersonal violence injury and connect them with the necessary resources to target their risk factors and promote their wellbeing (Carnell et al. 2006). HVIPs have been shown to reduce youth violence recidivism and reinjury (Butts and Delgado 2017; Becker et al. 2004). Project Ujima is an HVIP that is sponsored by Children’s Wisconsin (CW) and serves Milwaukee youth presenting to the Emergency Department/Trauma Center (EDTC). The program targets youth aged 7–18 years old being treated for violence-related injuries, excluding child abuse, self-inflicted injuries, and sexual assault, and provides crisis intervention, case management, and community resources. After a patient is referred, they are visited by HVIP staff, typically in the EDTC or during their hospitalization, to provide immediate crisis intervention, discuss program services, and obtain consent to be enrolled in the program.

Enrollment into Project Ujima is completely voluntary. Ideally, every youth who comes to the EDTC with violence-related injuries and meets the program’s criteria would be referred and enrolled. However, at our pediatric EDTC, the baseline referral rate to the HVIP was 32.5%, which demonstrates we are not effectively reaching a large portion of our target population. We propose that we can increase the utilization of our HVIP’s services by determining the eligibility of youth with violent injuries in our EDTC and implementing key interventions to increase the number of referrals. We aim to 1) determine the number of patients aged 7–18 years old who present to our EDTC with a violence-related injury that are eligible for our HVIP’s services; 2) increase the percent of eligible patients referred to our HVIP’s services from 32.5 to 70% within 12 months.

## Methods

This quality improvement project was undertaken between 11/1/2018 and 11/30/2019, with baseline data collected between 11/1/2018 and 3/1/2019. Our quality improvement team included medical students, pediatric emergency medicine physicians, staff from our HVIP, and a research analyst. Our target population of patients were youth aged 7–18 years who had ICD 10 code diagnoses consistent with injuries related to assault, stabbing, or gunshot wounds. The following patients are ineligible for our HVIP’s services: 1) Patients with injuries related to sexual assault, violence between family members, child maltreatment, or self-inflicted injuries, 2) patients who reside in group homes or outside our HVIP’s county, 3) patients with duplicative services. Patients who meet eligibility criteria for our HVIP are identified by emergency department staff (physicians, triage nurses, physician assistants), who either consult our HVIP or social workers to further assess the patient’s situation and eligibility. Subsequently, two medical students reviewed the complete record to assess: 1) eligibility for program services and 2) documentation of Project Ujima referral or intervention. At the outset of the record review, an emergency medicine physician (MML) performed a concurrent review of five records with each student to assure agreement.

We performed process mapping of the referral process that included 1) identifying eligible patients within our target population using our HVIP’s criteria and 2) recording when referrals were placed in relation to the ED visit and which EDTC staff placed them (i.e. nurses, physicians, social workers) and 3) recording when our HVIP staff contacted referred patients and 4) recording whether referred patients consented to the HVIP. One author (MML) interviewed key stakeholders, including nurses, social workers, and providers, who helped identify challenges to placing referrals and ways to intervene. From notes taken during these interviews, themes were compiled which included: 1) understanding Project Ujima eligibility and 2) concerns about timeliness of the Project Ujima consult and whether families wait for the Project Ujima advocate to come to the EDTC and 3) ability of nursing staff to enter a consult order and 4) understanding what Project Ujima services include and the subsequent patient outcomes after specific services.

We used Plan-Do-Study-Act (PDSA) cycles to inform our project. The study consisted of a pre-intervention period from 11/1/2018–2/28/2019, an intervention period from 3/1/2019 to 6/30/2019, and a post-intervention period from 7/12019–11/30/2019. Our key interventions, which were implemented by EDTC physicians and HVIP staff, consisted of the following:

1. Educating Pediatric Emergency Department providers on eligibility requirements (March 2019)
2. Providing accessibility for nurses to enter consults (April 2019)
3. Encouraging nurses to enter consults at the time of admission in triage (April 2019)
4. Publishing information about program referrals in the weekly nursing newsletter (May 2019)
5. Updating social workers on eligibility requirements for the HVIP (June 2019)

Educating providers and team members consisted of delivering an in-person presentation about our HVIP’s services, eligibility, and outcomes. It was followed by a question-and-answer session as well as time to suggest areas to improve the referral process. Separate sessions were held with EDTC social workers and the Nursing Council, comprised of nurses who represent all EDTC shifts.

Our primary outcome measure was the number of patients referred to our HVIP and all measures were analyzed using statistical process control charts. Our quality improvement project was exempt from IRB review.

## Results

Demographics are listed in Table 1. For our target population, those age 7–18 years who present to the EDTC with intentional violent injuries, 37.8% were female and the average age was 14.4 ± 2.6 years. Assault injuries (83.5%) were most common, while 16.5% sustained gunshot wounds. 14.9% of patients were hospitalized for their injuries. Within the HVIP-eligible population, 31.1% were female and the average age was 14.3 ± 2.7 years. Assaults represented 82.6% of injuries while gunshot wounds represented 17.4%.

**Table 1:** Demographics: Target Group vs. HVIP-eligible Group. Target Group represents all patients aged 7–18 years presenting to the EDTC with intentional violent injuries

Demographics: Target Group vs. HVIP-eligible Group		
	Target Group (*n* = 249)	HVIP-eligible Group (*n* = 167)
Female (n, %)	94, 37.8%	52, 31.1%
Age, years (mean, [SD])	14.4 [2.6]	14.3 [2.7]
Injury type		
Assault (n, %)	208, 83.5%	138, 82.6%
Gunshot wound (n, %)	41, 16.5%	29, 17.4%
Hospitalized (n, %)	37, 14.9%	24, 14.4%
Consult placed by		
Social Work	40, 48.8%	39, 50.0%
Nurse	15, 18.3%	14, 17.9%
Provider	27, 32.9%	25, 32.1%

From 11/2018 to 11/2019, there were 167 patients eligible for our HVIP out of 249 total patients (Table 1). Within our HVIP-eligible population, there were 78 patients referred. Chi-squared analysis showed a significant difference between the number of referrals placed between the pre-intervention and post-intervention periods (χ2 = 7.5275, *p* = 0.006). During the pre-intervention period, 13 of the 40 eligible patients (32.5%) were consulted to the HVIP. From July 2019 to November 2019, the post-intervention period, 33 of the 54 eligible patients were consulted to the HVIP, resulting in an increase to 61.1%. While social workers placed 50.0% of all referrals, nurses placed 17.9% and providers (i.e. physicians, physician assistants, nurse practitioners) placed 32.1% (Table 1).

**Fig. 1:**
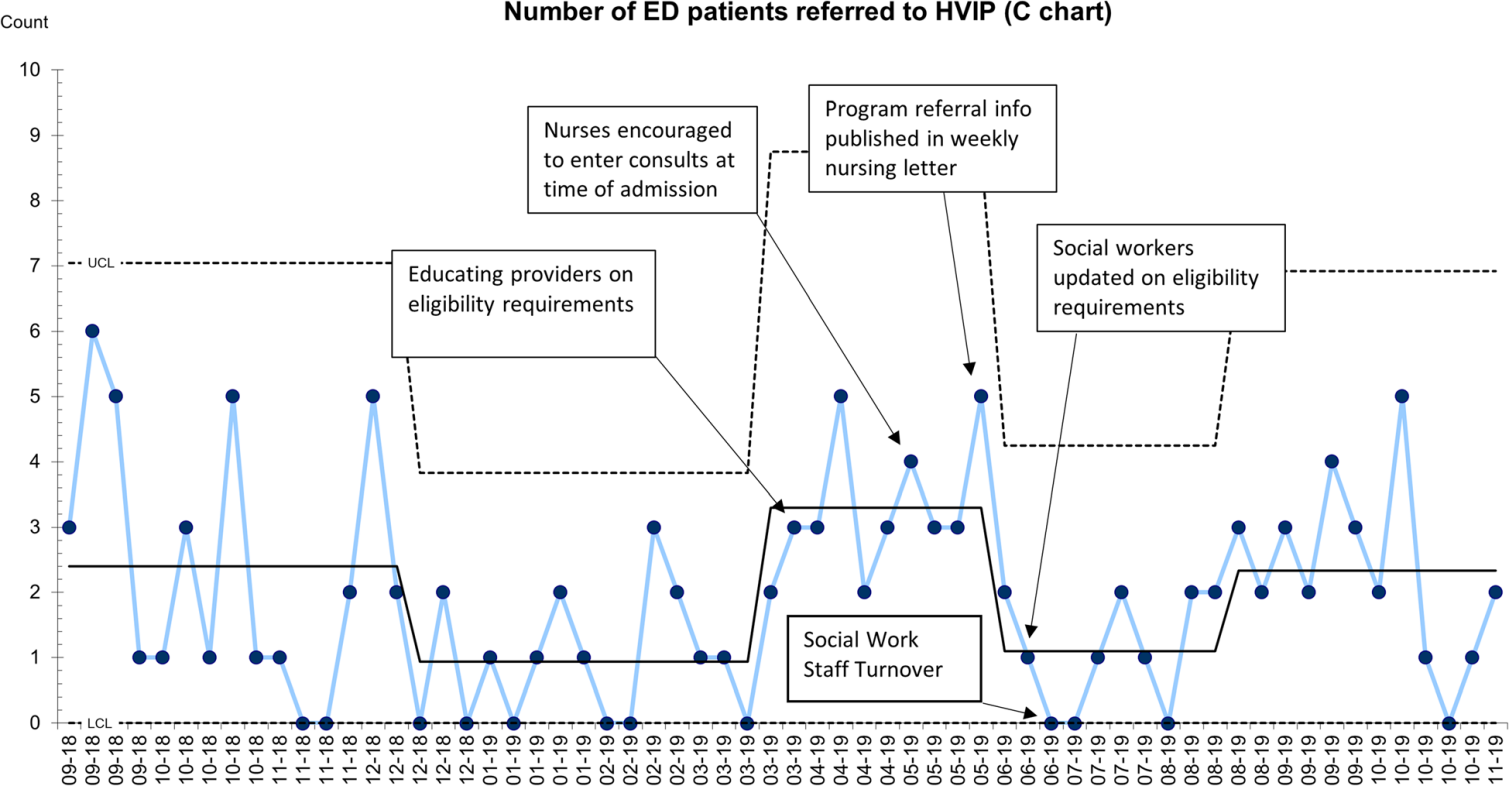
Number of ED patients referred to HVIP (C-chart). Upper control limit (UCL) and lower control limit (LCL) represent +/− 3 standard deviations from the centerline

Our first intervention consisted of educating providers on eligibility requirements with no observable effects seen in the referral process regarding counts within the first month. However, after a month of intervention, our referrals increased to referral counts at 5. Our next intervention consisted of enabling nursing staff to enter consults as well as inclusion criteria of the HVIP being placed in the nursing newsletter, the nursing triage area, and the social work office. The most significant results were seen regarding education amongst nursing staff with referral counts reaching their highest at 7 (Fig. 1). Nursing staff referrals included but were not limited to assessing inclusion eligibility based on patient chief concern, HPI findings, as well as chart review. There was an observable decline in referrals following education amongst social workers. With social work turnover occurring immediately after this intervention, this likely contributed to the lack of sustainability in results.

## Discussion

The goal of our project was to increase the number of referrals of eligible patients to Project Ujima from a baseline of 32.5 to 70%. To achieve this goal, we identified patients eligible for our HVIP, interviewed key stakeholders in the referral process, and implemented several educational interventions to EDTC healthcare providers. We observed an increase in the percent of eligible patients referred to our HVIP from 32.5 to 61.1%. However, this initial improvement was not sustained. Youth violence is a public health concern with high rates of recidivism; therefore, we recognize the need to correctly identify at-risk youth and refer them to our HVIP’s services.

There are a few reasons that likely contributed to why we did not meet our goal to increase referrals to 70%. Social workers have an important role in assessing the eligibility of patients and explaining our HVIP to them and their families. They are consulted by medical providers to gather more history related to the injury, assess the needs of the patient and family, and determine how to best meet those needs. They are responsible for documenting their assessments of patients as well as the details of the conversation regarding our HVIP (i.e. if materials were provided, if patients or families are interested in talking with an HVIP representative or not, if further elicited history makes the patient ineligible). Social worker turnover occurred near the end of our intervention period, which may have contributed to fewer referrals. Furthermore, many of the social workers who staff the ED in the evenings and overnight work part time and may have other daytime positions. Several of them had the opportunity for other positions and so left the institution. Additionally, one of the full-time social workers retired. So, in a short time, following our training, there were many new social workers staffing during the evening, when many of the violently injured youth present.

We also recognize that the EDTC is a high-stress environment for patients and their families. Receiving information about an HVIP in these moments may not be ideal for every family, causing them to refuse our HVIP’s services before a referral is placed. There are potentially many reasons why patients and families decline our HVIP’s services. However, because these instances are not clearly or routinely documented in provider or social work notes, it is difficult to collect this information. Finally, the EDTC is also a high-stress and fast-paced environment for providers, who may not always remember to identify eligible patients and place referrals.

Future studies will focus on analyzing and implementing additional interventions to increase referrals. One example includes implementing best practice alerts. The referral process to our HVIP uses the EDTC’s electronic health record (EHR). Best practice alerts (BPAs) are a growing tool that have been shown to improve health outcomes and reduce costs for multiple diseases and patient populations (Swedlund et al. 2019; Bejjanki et al. 2018). Implementing best practice alerts may help healthcare providers appropriately screen and place consults for our HVIP or even allow for automation of the referral process (Devoe et al. 2019). Now that we have standard criteria for identifying eligible patients, we can use it to screen for youth who present to our EDTC with violent injuries as we develop a BPA.

Additional interventions will focus on increasing the awareness of our HVIP within the EDTC, such as departmental gratitude messages to healthcare providers who place consults and increased reminders to EDTC staff to place consults. We will also continue to analyze factors in a patient’s ED visit timeline that may influence successful referral and enrollment, such as total time between when patients present and when they are contacted by the HVIP staff, who is present when patients are consulted, and if referrals are placed during or after the admission. In analyzing these factors, we plan to correlate referrals with daily EDTC flow and seasonal variations. Hopefully, understanding these components will allow us to make impactful changes to the referral process that increase the number of youths enrolled in our services.

There are a few limitations to our project. One is the limited pre- and post-intervention study time. Perhaps we require a more longitudinal study to observe a sustained improvement in referrals to our HVIP. An additional limitation is that we measured the percentage of referrals after clustering our interventions within a few months. We may have found that particular interventions were more impactful than others if we included more time between interventions or repeated interventions for the multiple EDTC shifts. Also, we were limited in our ability to assess barriers to successful referral placement or correlate referrals with daily EDTC flow or seasonal variations. Finally, as we conducted chart review to obtain our data, our ability to determine eligibility was susceptible to any potential limitations of the documentation in the electronic health record.

## Conclusions

In this quality improvement project, we were able to determine the eligible population as well as identify areas to intervene in our EDTC to improve the referral process for our HVIP, Project Ujima. After implementing these interventions, we analyzed whether they had a significant impact on the number of referrals. Future studies will examine additional interventions to create a sustained improvement in referrals to our HVIP, which will hopefully lead to increased utilization of services and decreased youth violence rates.

## Data Availability

Data sharing is not applicable to this article as no datasets were generated or analyzed during the current study.

## Abbreviations

BPAs: Best practice alerts
CW: Children’s Wisconsin
EDTC: Emergency Department/Trauma Center
EHR: Electronic Health Record
HVIPs: Hospital-based Violence Intervention Programs
PDSA: Plan-Do-Study-Act
